# Changes of Microrna Levels in Plasma of Patients with Rectal Cancer during Chemoradiotherapy

**DOI:** 10.3390/ijms18061140

**Published:** 2017-05-27

**Authors:** Peter Jo, Azadeh Azizian, Junius Salendo, Frank Kramer, Markus Bernhardt, Hendrik A. Wolff, Jens Gruber, Marian Grade, Tim Beißbarth, B. Michael Ghadimi, Jochen Gaedcke

**Affiliations:** 1 Department of General-, Visceral-, and Pediatric Surgery, University Medical Center Goettingen, Robert-Koch-Str. 40, 37075 Goettingen, Germany; jo.peter@chirurgie-goettingen.de (P.J.); azadeh.azizian@med.uni-goettingen.de (A.A.); juniussalendo@gmail.com (J.S.); markus.bernhardt@med.uni-goettingen.de (M.B.); marian.grade@med.uni-goettingen.de (M.G.); mghadim@uni-goettingen.de (B.M.G.); 2 Department of Medical Statistics, University Medical Center Goettingen, Robert-Koch-Str. 40, 37075 Goettingen, Germany; frank.kramer@med.uni-goettingen.de (F.K.); tim.beissbarth@med.uni-goettingen.de (T.B.); 3 Department of Radiology, Nuclear Medicine and Radiotherapy, Radiology Munich, Burgstr. 7, 80333 Munich, Germany; drhawolff@googlemail.com; 4 German Primate Center, Medical RNA Biology, Kellnerweg 4, 37075 Goettingen, Germany; jgruber@dpz.eu

**Keywords:** microRNA, rectal cancer, neoadjuvant chemoradiotherapy, plasma, tumor biopsy, liquid biopsy, biomarkers, prognosis

## Abstract

Since the response to chemoradiotherapy in patients with locally advanced rectal cancer is heterogeneous, valid biomarkers are needed to monitor tumor response. Circulating microRNAs are promising candidates, however analyses of circulating microRNAs in rectal cancer are still rare. 111 patients with rectal cancer and 46 age-matched normal controls were enrolled. The expression levels of 30 microRNAs were analyzed in 17 pre-treatment patients’ plasma samples. Differentially regulated microRNAs were validated in 94 independent patients. For 52 of the 94 patients a paired comparison between pre-treatment and post-treatment samples was performed. miR-17, miR-18b, miR-20a, miR-31, and miR-193a_3p, were significantly downregulated in pre-treatment plasma samples of patients with rectal cancer (*p* < 0.05). miR-29c, miR-30c, and miR-195 showed a trend of differential regulation. After validation, miR-31 and miR-30c were significantly deregulated by a decrease of expression. In 52 patients expression analyses of the 8 microRNAs in matched pre-treatment and post-treatment samples showed a significant decrease for all microRNAs (*p* < 0.05) after treatment. Expression levels of miR-31 and miR-30c could serve as valid biomarkers if validated in a prospective study. Plasma microRNA expression levels do not necessarily represent miRNA expression levels in tumor tissue. Also, expression levels of microRNAs change during multimodal therapy.

## 1. Introduction

Treatment of locally advanced rectal cancer has changed in recent years by introducing preoperative chemoradiotherapy (CRT) [1]. Extensive histopathological work-up of the tumor specimen after surgery including tumor regression grading (TRG) [2] and lymph node status (ypN) helped to visualize individual tumor sensitivity to CRT retrospectively. As this translates into patients’ prognosis [3] many research groups focus on the identification of prognostic or predictive parameters to enable an individualized risk-adopted therapy. A relevant number of studies aimed to identify molecular markers retrieved from tumor tissue while the relevance of blood-based biomarkers is less stringent assessed. Blood samples, however, offer several advantages: first, while taking biopsies is an uncomfortable, invasive procedure for patients, which is not without clinical complications [4], taking blood samples is less invasive, less expensive, easy to schedule, and nearly without any severe complications. Second, sample preservation and intratumoral heterogeneity limit the informative value of tumor biopsies for molecular analysis [5]. Especially in the case of rectal cancer, beyond intratumoral heterogeneity, tumor biopsies are in general accompanied by normal, adenomatous or stromal tissue. This contamination may affect results of molecular analyses [6,7]. Resorting to bloodstream bypasses those problems: blood samples are a source of fresh DNA and RNA, without modifications due to preservatives. Additionally, investigating blood from patients can account for molecular heterogeneity and surrogate for tumor burden since tumor-derived fragments or biomarkers are collected from all tumor cells in a patients’ body through circulation [8]. Therefore, they may offer both the possibility of dynamic monitoring under treatment and the possibility to assess disease activity even after pathologic complete response (pCR) or after resection of the tumor when no tissue is left for molecular analyses. In summary, blood is a promising biomaterial that should be analyzed aiming to identify biomarkers that could help to reveal tumor occurrence, identify molecular characteristics of the tumor and stratify cancer treatment.

In clinical routines to date carcinoembryonic antigen (CEA) is established as a colorectal cancer (CRC) related tumor marker but is not recommended as a screening test for colorectal cancer [9]. First, normal levels of CEA do not exclude the possibility of a colorectal cancer. Second, an elevated CEA is not categorically associated with CRC, or in the period of follow-up with disease progression. In rectal cancer there are several studies analyzing changing CEA levels in relation to preoperative CRT. High CEA levels pre- as well as post-therapeutical were associated with a poorer prognosis [10,11,12,13,14,15,16]. However, the clinical use of CEA as a molecular biomarker to predict pathologic complete response has its limitations and is controversially discussed [11,17].

As a potential alternative to CEA, microRNAs (miRNAs) are currently under investigation to serve as blood-based biomarkers. miRNAs are small, noncoding RNAs that regulate gene expression by post-transcriptional mRNA binding, which promotes the destabilization of target mRNAs. The target specificity of miRNAs is largely predetermined by their so-called “seed-sequence” (containing nucleotides at position 2–7 of the miRNA). They are highly conserved between species, stable and easy detectable even in small concentrations. In the meantime a large panel of studies have indicated a role as circulating biomarker. They have been widely analyzed in physiological and pathological processes. Besides, miRNA expression is tissue specific [18]. Several sets of miRNAs are differentially up- or downregulated in tumors of different origins, although on the other hand, the miRNA signature of different cancer types can share individual miRNAs [19]. Considering the organ specificity of miRNAs and the particular treatment modalities of rectal cancer, it is a need to analyze blood samples from these patients. To date, no screening approach to identify relevant miRNAs as biomarkers in blood of patients with rectal cancer was undertaken. Hypothesizing that cancer-specific circulating miRNAs found in the blood of patients are associated to the individual tumor, we analyzed a subset that we have previously identified as tumor specific by profiling locally advanced rectal cancer tumor tissue and adjacent normal mucosa [20,21]. To investigate the role of circulating miRNAs as biomarkers the study was divided into two phases: in a discovery/selection (phase I) up- and downregulated miRNAs from the previous work were evaluated and in a validation phase (phase II) differentially regulated miRNAs were validated.

## 2. Results

### 2.1. Initial Selection of Differentially Regulated miRNAs in Rectal Cancer Patients Compared to Controls—Phase I

**Figure 1:**
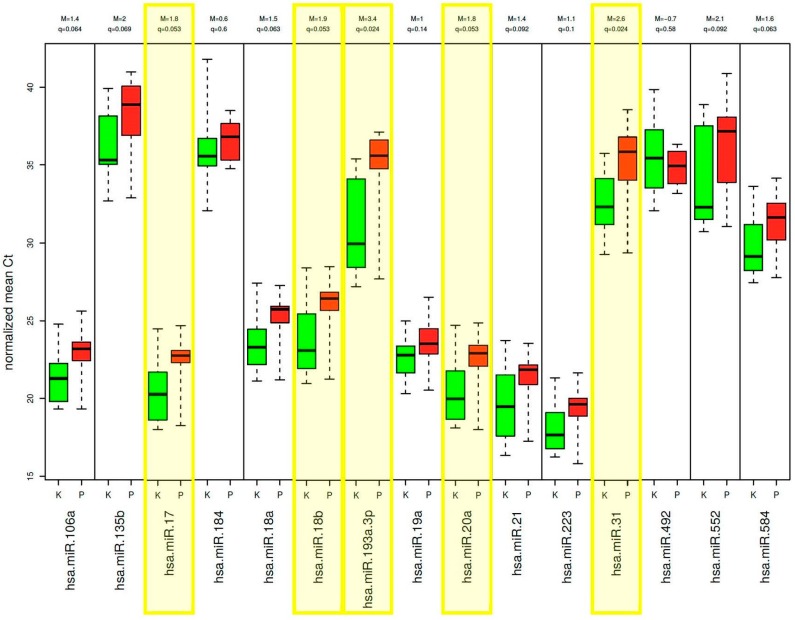
Expression levels of the 15 miRNAs, which were upregulated in tumor tissue compared to normal mucosa tissue, show a decreased expression level in plasma in comparison to the control samples, phase I. Boxplot of the 5 significant deregulated plasma miRNAs highlighted in yellow (red: patients with rectal cancer, green: healthy control group).

**Figure 2:**
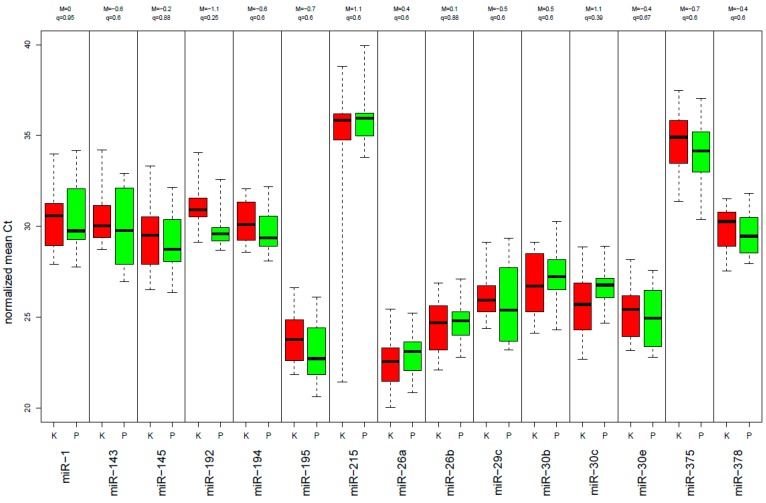
Expression levels of the 15 miRNAs, which were downregulated in tumor tissue compared to normal mucosa tissue, show no significant different expression level compared to the control group (red: rectal cancer patients, green: healthy control group).

Demographic and clinical information to this test-set, including 17 rectal cancer patients and 14 control samples, is presented in Supplementary Table S1. Among the 30 miRNAs that have been analyzed, 6 were excluded due to insufficient detection rate (50% of the *C*_t_ values were above a *C*_t_ value of 36; miR-135b, miR-184, miR-492, miR-552, miR-375 and miR-215). Interestingly, the expression levels of the 15 miRNAs, which were upregulated in tumor tissue compared to normal mucosa tissue, showed a decreased expression level in plasma of rectal cancer patients in comparison to the control samples. Five of them were significantly downregulated (*p* < 0.05): miR-17, miR-18b, miR-20a, miR-31, and miR-193a_3p (Figure 1). Of the 15 miRNAs that were downregulated in the tumor, none was significantly differentially expressed compared to the control group (Figure 2), however miR-29c, miR-30c and miR-195 at least showed a trend.

### 2.2. Validation of Differentially Expressed miRNAs—Phase I

**Figure 3:**
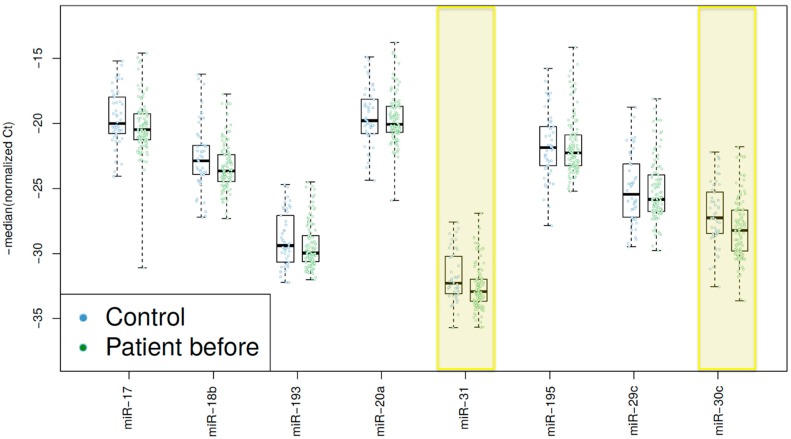
Boxplot showing the plasma expression levels of miRNAs in the control-group (Control) and in the patients with rectal cancer before neoadjuvant chemoradiotherapy (Patient before). miR-31 (*p* = 0.013) and miR-30c (*p* = 0.017) were significantly deregulated by a decrease of expression in the plasma of patients with rectal cancer.

To validate the identified miRNAs, an independent set of 94 rectal cancer patients and 32 control samples was analyzed. The clinical data of this validation set is presented in Supplementary Table S2. The five significantly downregulated miRNAs of the first set (miR-17, miR-18b, miR-20a, miR-31, and miR-193a_3p) were selected for further validation. Since all of them belong to the group of upregulated miRNAs in tumor tissue compared to mucosa, we included three additional miRNAs from the group of initially downregulated miRNAs in tumor tissue, which also showed a trend of differential regulation in blood of patients compared to normal controls: miR-29c, miR-30c and miR-195. Overall, eight miRNAs were selected for further investigation. Of these, miR-31 (*p* = 0.013) and miR-30c (*p* = 0.017) were significantly deregulated by a decrease of expression in the plasma of rectal cancer patients (Figure 3).

### 2.3. miRNA Expression-Level Changes during Multimodal Therapy

**Figure 4:**
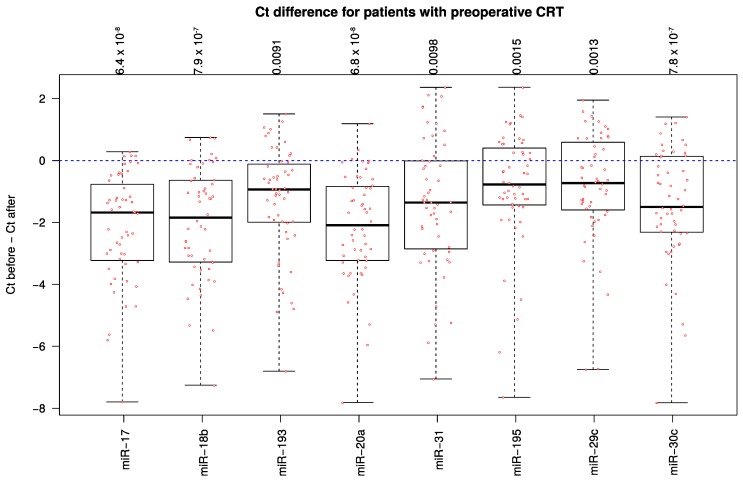
Boxplot showing the differences between miRNA expression levels before neoadjuvant chemoradiotherapy and after completion of therapy (after surgery) in patients with rectal cancer. *C*_t_ before–*C*_t_ after: Expression levels of the miRNA before treatment substracted to the respective miRNA expression levels after the completed treatment.

For 52 patients analyses of the 8 selected miRNAs were conducted comparing the expression-level changes before neoadjuvant chemoradiotherapy and after completion of therapy, 6–12 months after surgery. Interestingly, the expression in the plasma was significantly decreased for all eight miRNAs (*p* < 0.05) (Figure 4).

### 2.4. High Decrease of miRNA Expression Reveals A Trend for Better Prognosis

**Figure 5:**
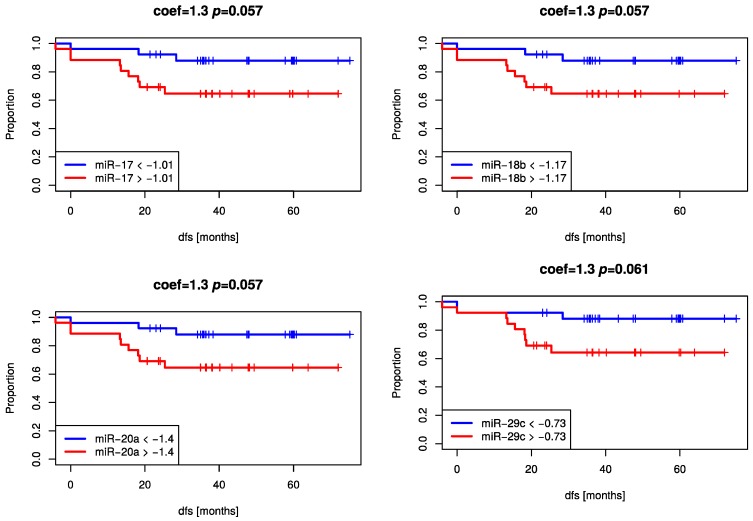
Kaplan–Meier Curves showing a trend for better DFS for plasma expression level changes between two time points (pre-CRT and post-adjuvant-CT) of miR-17, miR-18b, miR-20a (tumor-upregulated miRNAs) and miR-29c (tumor-downregulated miRNA).

To examine the prognostic role of miRNAs in the blood of rectal cancer patients, expression levels of the eight selected miRNAs from both time points were correlated to clinicopathological parameters (ypN, TRG, Disease Free Survival (DFS)). None of the miRNAs was associated with these relevant parameters. However, using the change of miRNAs-expression levels miR-17, miR-18b, miR-20a, and miR-29c at least showed a trend for better DFS in patients with higher decrease (Figure 5).

## 3. Discussion

The application of miRNAs in the blood of patients as potential biomarker is not new. However, in rectal cancer there are very little data [22] and due to the therapy differences and the anatomic localization of the rectum a simple transfer of data from colon to rectum is inappropriate. Accordingly we screened for the expression of miRNAs in the plasma of patients with locally advanced rectal cancer who were treated by preoperative chemoradiotherapy. The main focus of this study was the identification of miRNAs that are differentially expressed in cancer patients compared to healthy controls. The selection of miRNAs was based on a previous tumor and normal mucosa screen–a strategy that has been performed before [23]. Interestingly, all miRNAs that were retrieved from the group of upregulated miRNAs in the tumor showed a trend towards a reduced expression in the plasma of rectal cancer patients compared to the control samples. In contrast, expression levels of miRNAs in the plasma that were selected based on a decreased expression in the tumor compared to the mucosa were irregularly up- or downregulated miRNAs. Discrepant expression levels between plasma and tissue have been shown before. Xu et al. [24] screened the expression of miRNAs in colorectal cancer tissue and plasma compared to healthy controls. Of these, five miRNAs were validated and opposing expression levels were reported as well. Interestingly, all five miRNAs that were used for validation were downregulated in the tissue. Wulfken et al. [25] found a comparable discrepancy of alteration between circulating miRNAs and tissue miRNAs. Analyzing renal cell carcinoma 109 circulating miRNAs were at higher levels in the plasma but only 36 miRNAs were upregulated in the corresponding tissue samples. The authors concluded that obviously only a subset of circulating miRNAs have tumor-specific origins. The finding of reduced plasma concentration appears anti-intuitive. Pigati et al. [26] identified this phenomenon for breast cancer and postulated the reduction as retention of the miRNAs within the tumor cells. Although not proven the data were interpreted as if cells have a mechanism in place to select specific miRNAs for cellular release or retention. Yet the mechanisms of decreased miRNA expression levels in cancer plasma samples is still not investigated in detail and has to be elucidated in further experiments to reveal the complex mechanisms. In addition, decreased miRNA expression levels in cancer plasma samples might be rather a non-tumor-specific effect. Furthermore, normalization effects or sample quality could also influence a change of miRNA expression. In this context, we have to emphasize that each plasma sample was taken, stored and processed under stringent biobanking Standard Operating Procedures [7] since translational research relies on high-quality biospecimens.

After validation miR-31 turned out to be significantly downregulated in the plasma–in contrast to the tissue. Very recently Wang et al. [27] analyzed this and other miRNAs in the serum of a mixed colorectal cancer cohort and also described a lower expression in the tumor group compared to healthy controls. The expression pattern is not consistent over cancer of the gastrointestinal tract. While miR-31 was shown to be upregulated in colorectal cancer (CRC) a decreased expression was described in gastric [28] or pancreatic cancer [29]. In-vivo, up-regulation of miR-31 in CRC cells leads to increased cell proliferation, invasion, and metastasis [21,28]. In-vivo miR-31 directly represses SATB2 [30]. Sun et al. [31] demonstrated the activation of the RAS pathway by miR-31. Recently miR-31 has been shown to play a role in modulating radioresistance in esophageal adenocarcinoma, potentially enhanced via DNA repair and was tested as predictive marker for response of preoperative RCT [32]. Suppression of microRNA-31 increases sensitivity to 5-FU at an early stage [30].

In the group of downregulated miRNAs from the tumor tissue screen, finally miR-30c turned out to be significantly lower expressed in the plasma compared to healthy controls. As a member of the miR-30 family, very recently several groups have shown its tumor suppressive function in CRC [32,33,34,35] as well as other entities [36]. However, Wang et al. [37] showed opposing results in ovarian cancer.

Overall, miR-30c and -31 may have a potential relevance as biomarker in rectal cancer to distinguish between cancer and non-cancer patients in the plasma. However, these data need a larger validation as the biological explanation is still under debate. As mentioned above, the hypothesis of miRNA retention is possible. However, considering the fact of discrepant attitude towards the existence of a hormone-like function of miRNAs [38] and the absence of reliable data on an active uptake of specific miRNAs into the tumor, there is currently only a validated correlation. To further discuss this hypothesis of miRNA uptake by tumor cells we performed the analysis of plasma from patients after the tumor has been resected and the adjuvant therapy was administered; in conclusion a patient considered as tumor free. Certainly, there was the potency of potential dorming cells, however, none of the patients developed recurrence within 3 months after the time point of tumor free diagnosis. Accordingly, the absence of tumor should lead to an increase of circulating miRNAs, however, our findings showed again a decrease of expression. In this constellation, all of the miRNAs showed a significant change. To which extent these effects are tumor driven or present an effect of non-tumor processes that regulate the miRNA appearance in the blood is unclear. To answer this question, comparative studies using comparable treatment strategies are needed. Specifically, rectal cancer patients without preoperative treatment as well as patients with e.g. prostate cancer receiving radiotherapy need to be analyzed. A change of miRNA expression levels under radiotherapy has already been shown [39,40]. Although these data are not retrieved from patients the relevance of non-tumor specific effects need to be acknowledged. As it is well known, patients respond with different type and grade of toxicity on radiotherapy. Therefore, these effects may have an input on blood-based biomarkers.

Finally, it needs to be mentioned, that only a few members of the miR-17-92 cluster were found as significantly regulated. In particular, miR-92 was identified to drive oncogenesis in colon cancer [41] and was not among the deregulated miRNAs in plasma samples. This may indicate a general upregulation of the miR-17-92 polycistron in cancer progression, while only parts of the primary transcript are cell retained.

Although our results are promising, there are several limitations in this study. First, as the collective size is still small and miRNA plasma level changes show just a trend for a better prognosis, further validations in a larger cohort and in independent studies are necessary to confirm potential statistically significance. Second, the amount of some miRNAs (*n* = 6/30) in plasma are too low to be accurately quantified by failing the linear range of the assay, therefore, some potential relevant markers could not be considered for further analyses. Third, the investigated miRNAs are based on a previous study where tumor tissue and matched mucosa were analyzed. To avoid limitations in identifying relevant miRNA expression patterns for rectal cancer patients in plasma genome wide miRNA screen should be considered in blood samples in future.

## 4. Materials and Methods

### 4.1. Patients, Patient Treatment and Control Group

Overall, 111 patients with locally advanced rectal cancers treated in the Department of General, Visceral and Pediatric Surgery, University Medical Center Goettingen, Germany and 46 age-matched normal controls were enrolled in this study. All patients were enrolled in the CAO/ARO/AIO-94 [1] or the CAO/ARO/AIO-04 [42] trial (EudraCT-Number 2006-002385-20-NCT00349076) of the German Rectal Cancer Study Group. Accordingly, pretherapeutical assessment of the tumor was performed by endorectal ultrasound, computed tomography scan and magnetic resonance imaging. Staging results were described as clinically assessed T-level (cT), lymph node status (cN), distant metastases (cM), and UICC stage (cUICC). All patients received a preoperative chemoradiotherapy (CRT): Total radiation dose was 50.4 Gray (Gy) in 28 fractions accompanied by either an intravenous (iv) application of 5-FU (1000 mg/m^2^ on days 1–5 and 29–33) or a combination of an iv-infusion of oxaliplatin and 5-FU according to the study protocol. Six weeks after the completion of preoperative CRT, curative total-mesorectal-excision-surgery (TME) was performed. Four to six weeks after surgery treatment was completed with an adjuvant therapy with either 5-FU or 5-FU with folinic acid combined with oxaliplatin. Blood was taken prior to preoperative CRT and after completion of adjuvant therapy. At the second time point patients had no signs of disease progression and accordingly were considered as cancer free. Blood samples for the control group (*n* = 32) were obtained from patients treated for either groin hernia or asymptomatic cholecystolithiasis. Further inclusion criteria were age between 60 and 80, no malignant disease in the past, colonoscopy without pathological findings within the last four years, no clinical signs of acute inflammation and unremarkable laboratory parameters (blood count, coagulation, liver enzymes). Written informed consent was obtained from all patients. This study conformed to the ethical principles of the Declaration of Helsinki (Seoul, Korea, 2008) and was approved by the University of Goettingen Ethics Committee in Goettingen, Germany (application number 20/9/95, 9/8/08).

### 4.2. Study-Design

**Figure 6:**
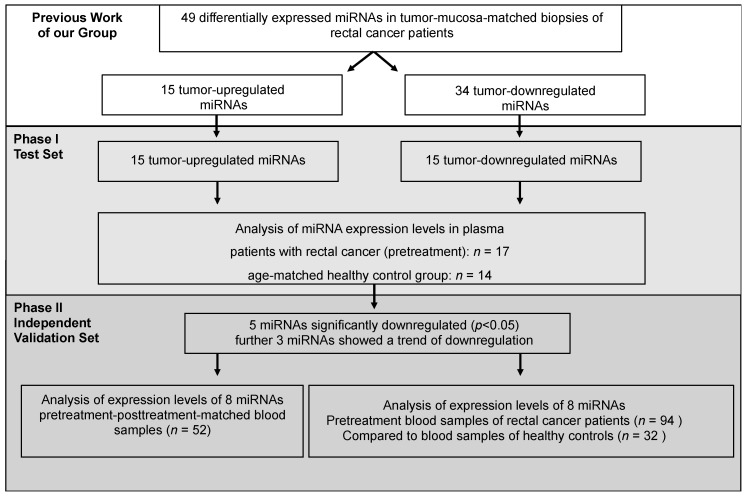
Study-design. The results of our previous work (Gaedcke et al, 2012) [20] showed 49 differentially expressed miRNAs in tumor-mucosa-matched biopsies of patients with rectal cancer. Of those 49 miRNAs 15 were upregulated in tumor and 34 were downregulated. Phase І: In the present work we analyzed in blood samples the expression levels of the 15 tumor-upregulated miRNAs and also 15 of the 34 tumor-downregulated miRNAs. In conclusion, expression levels of 30 miRNAs in blood samples of 17 rectal cancer patients were compared to the blood samples of normal controls (*n* = 14). Phase II: Subsequently, the differentially expressed miRNAs from the first set were analyzed in an independent validation set. Also, the expression levels of the 8 miRNAs were compared in the blood of patients with rectal cancer in matched pre-treatment and post-treatment samples.

The study was divided into two phases: a discovery/selection (phase І) and a validation phase (phase II) (Figure 6). In a previous work of our group a tissue-based comparison of paired tumor and normal mucosa samples of patients with locally advanced rectal cancer identified 49 differentially expressed miRNAs [20]. Of those 15 were upregulated while 34 were down-regulated in the tumor tissue samples compared to the paired mucosa samples.

Phase І: Expecting a higher likelihood for upregulated miRNAs to be found in blood, all of the 15 upregulated miRNAs were selected, while only 15 of the 34 downregulated miRNAs were chosen for the initial discovery phase. These analyses were performed using 17 pre-therapeutical plasma probes of rectal cancer patients that were subsequently treated by CRT and 14 control samples.

Phase II: In the next step miRNAs that turned out to be differentially regulated between rectal cancer patients and the control group were validated. This phase comprised 94 independent patients and 32 control samples. Of importance, for 52 of the 94 patients a paired comparison between pretreatment (pre-CRT) and post-treatment (after surgery and adjuvant chemotherapy) samples, respectively, was possible.

### 4.3. Plasma Preparation and Total RNA Isolation

Plasma was used for miRNA quantification. Therefore, peripheral blood was drawn into a standard 7.5 mL EDTA tubes (EN 14280) (Sarstedt, Nuembrecht, Germany). Tubes were subjected to centrifuge at 2200 g at 4 °C for 10 min. Subsequently, 2 mL aliquots of the plasma were transferred into tubes and stored at −80°C. Prior to the analysis, the plasma samples were thawed and 250 μL was transferred to a 1.5 mL tube and centrifuged at 1000 g for 5 min to remove any visible cellular debris. Then, the supernatant was transferred into fresh tubes for further analyses.

The total RNA was extracted by using the miRNeasy Mini Kit (Qiagen, Hilden, Germany) according to the manufacturer’s protocol with the following modifications: The probes were lysed using 5× volumes Qiazol (Qiagen, Hilden, Germany). After 20 min incubation at room temperature, 3 synthetic mimics of *Caenorhabditis elegans* (cel-miR-39, cel-miR-54 and cel-miR-238) of each 12.5 fmol were added to the solution for the normalization process [43]. Further steps were done according to the manufacturer instructions and the total RNA was eluted in 40 μL purified water.

### 4.4. Semi-Quantitative Real-Time PCR Analysis

Semi-quantitative real-time PCR analyses were performed using miScript Qiagen Kit (Qiagen, Hilden, Germany) according to the manufacturer’s instructions. After reverse transcription using RT II Kit (Qiagen, Hilden, Germany), cDNA with universal tags were put in together with the specific miRNA Primer (see Table S3) and Master mix for the quantitative PCR reactions using Biorad CFX384 Real Time System (Bio-Rad Laboratories, Hercules, CA, USA). All assays were performed in triplicates. miRNA expression was presented as normalized *C*_t_ values, where *C*_t_ = threshold cycle, normalized *C*_t_ of sample X = (*C*_t_ target microRNA of sample X − average cel-miR-39, cel-miR-54 and cel-miR-238 of sample X + average cel-miR-39, cel-miR-54 and cel-miR-238 of all samples).

### 4.5. Data Analysis

In order to identify miRNAs that were differentially expressed, we applied the empirical Bayes, moderated *t*-statistics implemented using the R software package “limma” [44]. For disease-free (DFS) and overall survival (OS) analysis, Kaplan–Meier plots and the Cox proportional hazards model were applied using the R package “survival” [45]. DFS was defined as time from resection of the tumor (patients considered as tumor free) until the development of distant or local recurrence, and OS as time until tumor-related death. *p*-Values smaller 0.05 (*p* < 0.05) were considered significant. In order to not exceed a false discovery rate (*q*-value, Q) of 5%, *p*-values were adjusted for multiple testing using the Benjamini-Hochberg method [46]. All analyses were performed using the free statistical software R (version 2.14.1, the R Foundation for Statistical Computing, Vienna, Austria).

## 5. Conclusions

In conclusion, we could identify two differentially expressed miRNAs in plasma probes of rectal cancer patients based on a previous study comparing tumor tissue and matched mucosa samples. Hereby, plasma miRNA expression does not necessarily represent miRNA expression levels in tumor tissue. miRNA expression plasma changes between two time points, before and after multimodal therapy, could predict prognosis. Future efforts are still needed to identify circulating microRNA expression patterns that can accurately detect rectal cancer at an early stage and could serve as predictive and prognostic biomarkers.

## Supplementary Materials

Supplementary materials can be found at www.mdpi.com/1422-0067/18/6/1140/s1.

## Abbreviations

CEA	carcinoembryonic antigen
CT	chemotherapy
CRT	chemoradiotherapy
DFS	disease-free-survival
DNA	deoxyribonucleic acid
f	female
m	male
miRNA	microRNA
OS	overall-survival
pCR	pathologic complete response
PCR	polymerase chain reaction
RNA	ribonucleic acid
TME	total mesorectal excision
TRG	tumor regression grade
ypN	post-chemoradiotherapy nodal status
