# Supplementary material for: Changes of Microrna Levels in Plasma of Patients with Rectal Cancer during Chemoradiotherapy
Source: Int J Mol Sci. 2017 May 27;18(6):1140. doi: 10.3390/ijms18061140 (PMC5485964; doi:10.3390/ijms18061140)
Supplement: Supplementary file 1 [file ijms-18-01140-s001.pdf]

Supplementary Information

Table S1. Demographic and clinical information of the test-set including 17 rectal cancer patients, mean age 62.35, (* paired pretreatment and post-treatment samples, m = male, f = female, cT = initial clinical T-level, measured by ultrasound and MRI, cN = initial clinical nodal status, measured by ultrasound and MRI, cM = initial clinical metastasis status, determined by imaging techniques, RT = radiotherapy, TRG = tumor regression grade, distant met. = distant metastasis, local = local recurrence, NA = Not available).

Patient	Sex	Age	cT	cN	cM	Preoperative Therapy	TRG	Recurrence
P001	m	70	3	1	0	5-FU + RT	1	no
P002*	m	55	3	1	0	5-FU + Oxaliplatin + RT	2	local & distant met.
P003	f	81	3	1	0	5-FU + RT	2	no
P004	m	66	3	1	0	5-FU + Oxaliplatin + RT	3b	no
P005	m	59	3	1	0	5-FU + RT	3b	distant met.
P006	m	62	3	0	0	5-FU + RT	1	no
P007	f	58	3	1	0	5-FU + RT	3a	no
P008	f	68	3	1	0	5-FU + RT	2	no
P009	m	63	3	1	0	5-FU + RT	3	no
P010	f	76	3	0	0	5-FU + Oxaliplatin + RT	3b	no
P011*	m	65	3	1	0	5-FU + Oxaliplatin + RT	3b	no
P012*	m	56	3	0	0	5-FU + Oxaliplatin + RT	3a	no
P013	m	53	3	1	0	5-FU + Oxaliplatin + RT	3b	distant met.
P014	m	56	3	1	0	5-FU + RT	4	no
P015*	m	60	2	1	0	5-FU + RT	4	no
P016	f	63	3	0	0	5-FU + RT	2	local & distant met.
P017	m	49	3	0	0	5-FU + RT	1	distant met.

Table S2. Demographic and clinical information of the validation-set including 94 patients with rectal cancer, mean age 64.43, (* paired pretreatment and post-treatment samples, m = male, f = female, cT = initial clinical T-level, measured by ultrasound and MRI, cN = initial clinical nodal status, measured by ultrasound and MRI, cM = initial clinical metastasis status, determined by imaging techniques, RT = radiotherapy, TRG = tumor regression grade, distant met. = distant metastasis, local = local recurrence, NA = not available).

Patient	Sex	Age	cT	cN	cM	Preoperative Therapy	TRG	Recurrence
P018	m	54	3	1	0	5-FU + RT	3b	no
P019	m	72	3	1	0	5-FU + Oxaliplatin + RT	4	no
P020	m	57	3	1	0	5-FU + Oxaliplatin + RT	3b	no
P021	m	60	3	0	0	5-FU + RT	2	no
P022	f	66	3	1	0	5-FU + RT	3a	distant met.
P023	m	42	3	1	1	5-FU + Oxaliplatin + RT	2	distant met.
P024	m	79	3	1	0	5-FU + RT	3b	no
P025	m	65	3	1	0	5-FU + RT	2	no
P026	m	63	3	1	0	5-FU + Oxaliplatin + RT	3b	no
P027	m	76	4	1	0	5-FU + RT	2	no
P028	f	77	3	1	0	5-FU + Oxaliplatin + RT	2	no
P029	m	55	3	1	0	5-FU + RT	3b	local & distant met.
P030	m	61	3	1	0	5-FU + Oxaliplatin + RT	3a	no
P031	f	63	3	1	0	5-FU + RT	3b	distant met.
P032	f	36	3	1	0	5-FU + Oxaliplatin + RT	3b	distant met.
P033	m	63	3	1	0	5-FU + Oxaliplatin + RT	2	no
P034	f	64	3	0	0	5-FU + RT	2	no
P035	f	75	3	0	0	5-FU + RT	NA	no
P036	m	75	3	0	0	5-FU + Oxaliplatin + RT	3b	distant met.
P037	m	63	3	0	0	5-FU + RT	NA	no
P038	m	68	3	0	0	5-FU + Oxaliplatin + RT	2	distant met.
P039	m	61	3	1	0	5-FU + RT	3b	no
P040	f	55	3	1	1	5-FU + RT	NA	distant met.
P041	m	79	2	1	0	5-FU + RT	NA	no
P042	f	63	3	0	0	5-FU + RT	NA	no
P043	m	60	3	1	0	5-FU + Oxaliplatin + RT	3a	local & distant met.
P044	f	85	3	1	0	5-FU + RT	NA	no
P045	m	49	3	0	0	5-FU + RT	NA	no
P046	m	73	2	1	0	5-FU + RT	NA	no
P047	m	54	3	1	0	5-FU + RT	2	local & distant met.
P048	m	74	3	1	0	5-FU + RT	2	no
P049	m	60	3	1	0	5-FU + RT	4	no
P050	f	76	3	0	0	5-FU + RT	NA	distant met.
P051	m	65	4	0	0	5-FU + RT	3a	no
P052	m	68	3	0	0	5-FU + RT	NA	no
P053	m	62	4	1	0	5-FU + RT	2	no
P054	f	67	2	1	0	5-FU + RT	NA	distant met.
P055	m	71	3	0	0	5-FU + RT	NA	no
P056	f	67	4	1	0	5-FU + Oxaliplatin + RT	2	no
P057	m	81	3	1	0	5-FU + RT	NA	no
P058	m	75	2	1	0	5-FU + RT	NA	no
P059	f	69	3	1	0	5-FU + RT	NA	no
P060	m	63	4	0	0	5-FU + RT	2	distant met.
P061	m	73	3	0	0	5-FU + RT	NA	no
P062	m	63	3	0	0	5-FU + RT	NA	no

Patient	Sex	Age	uT	uN	cM	Preoperative Therapy	TRG	Recurrence
P063	m	75	3	1	0	5-FU + RT	NA	no
P064*	f	72	3	1	0	5-FU + RT	3b	no
P065*	m	65	3	1	0	5-FU + Oxaliplatin + RT	3b	distant met.
P066*	m	67	3	1	0	5-FU + RT	NA	no
P067*	m	79	3	1	0	5-FU + Oxaliplatin + RT	2	no
P068*	m	71	3	1	0	5-FU + RT	3b	no
P069*	m	66	3	1	0	5-FU + Oxaliplatin + RT	4	no
P070*	f	47	3	1	0	5-FU + Oxaliplatin + RT	3a	no
P071*	m	69	2	1	0	5-FU + RT	1	no
P072*	m	73	3	1	0	5-FU + RT	3b	no
P073*	m	53	2	1	0	5-FU + RT	3a	no
P074*	m	58	4	1	0	5-FU + RT	3a	no
P075*	m	45	3	1	1	5-FU + RT	NA	distant met.
P076*	f	68	2	1	0	5-FU + Oxaliplatin + RT	4	no
P077*	f	50	3	1	0	5-FU + Oxaliplatin + RT	1	local
P078*	m	77	3	0	0	5-FU + RT	NA	no
P079*	m	53	3	0	0	5-FU + RT	4	no
P080*	f	59	3	1	0	5-FU + Oxaliplatin + RT	3b	no
P081*	f	74	3	1	0	5-FU + RT	3b	no
P082*	f	69	3	1	0	5-FU + RT	NA	no
P083*	f	68	3	1	0	5-FU + Oxaliplatin + RT	2	no
P084*	m	66	4	1	0	5-FU + RT	4	no
P085*	m	61	3	1	0	5-FU + Oxaliplatin + RT	4	no
P086*	m	41	3	0	0	5-FU + Oxaliplatin + RT	4	no
P087*	m	76	3	1	0	5-FU + RT	2	distant met.
P088*	f	59	3	1	1	5-FU + Oxaliplatin + RT	3b	distant met.
P089*	m	68	3	0	1	5-FU + Oxaliplatin + RT	2	distant met.
P090*	m	57	3	1	0	5-FU + Oxaliplatin + RT	4	no
P091*	f	63	3	1	0	5-FU + Oxaliplatin + RT	3b	no
P092*	f	68	3	0	0	5-FU + RT	NA	no
P093*	m	61	3	0	0	5-FU + RT	NA	no
P094*	m	72	3	1	0	5-FU + RT	2	distant met.
P095*	f	71	3	1	0	5-FU + RT	3b	no
P096*	m	56	3	1	0	5-FU + RT	3	no
P097*	f	72	3	1	0	5-FU + RT	NA	no
P098*	f	51	3	1	0	5-FU + Oxaliplatin + RT	3b	no
P099*	f	56	3	1	0	5-FU + Oxaliplatin + RT	2	no
P100*	m	61	2	1	0	5-FU + RT	1	no
P101*	f	69	4	1	1	5-FU + RT	3b	distant met.
P102*	m	59	3	0	0	5-FU + RT	NA	no
P103*	m	69	3	0	0	5-FU + Oxaliplatin + RT	3b	no
P104*	f	63	3	1	0	5-FU + Oxaliplatin + RT	3b	no
P105*	m	53	3	1	0	5-FU + Oxaliplatin + RT	3b	distant met.
P106*	f	58	3	1	0	5-FU + RT	2	distant met.
P107*	f	52	3	0	0	5-FU + Oxaliplatin + RT	4	no
P108*	f	50	3	0	0	5-FU + RT	NA	no
P109*	m	75	2	1	0	5-FU + RT	NA	distant met.
P110*	m	77	3	1	0	5-FU + RT	3b	no
P111*	m	67	4	1	0	5-FU + Oxaliplatin + RT	2	no

Table S3. Target sequences for semi-quantitative real-time PCR analysis of the 15 upregulated and 15 downregulated miRNAs (tumor tissue vs mucosa tissue, investigated in a previous work – Gaedcke J, Clin Cancer Res, 2012).

miRNA	Target Sequence	Regulation in Tumor Tissue vs Mucosa (Tested in Previous Work)
miR-17	CAAAGUGCUUACAGUGCAGGUAG	Upregulated in tumor tissue
miR-18a	UAAGGUGCAUCUAGUGCAGAUAG	Upregulated in tumor tissue
miR-18b	UAAGGUGCAUCUAGUGCAGUUAG	Upregulated in tumor tissue
miR-19a	UGUGCAAAUCUAUGCAAAACUGA	Upregulated in tumor tissue
miR-20a	UAAAGUGCUUAUAGUGCAGGUAG	Upregulated in tumor tissue
miR-21	UAGCUUAUCAGACUGAUGUUGA	Upregulated in tumor tissue
miR-31	AGGCAAGAUGCUGGCAUAGCU	Upregulated in tumor tissue
miR-106a	AAAAGUGCUUACAGUGCAGGUAG	Upregulated in tumor tissue
miR-135b	UAUGGCUUUUCAUUCCUAUGUGA	Upregulated in tumor tissue
miR-184	UGGACGGAGAACUGAUAAGGGU	Upregulated in tumor tissue
miR-193a	AACUGGCCUACAAAGUCCCAGU	Upregulated in tumor tissue
miR-223	UGUCAGUUUGUCAAAUACCCCA	Upregulated in tumor tissue
miR-492	AGGACCUGCGGGACAAGAUUCUU	Upregulated in tumor tissue
miR-552	AACAGGUGACUGGUUAGACAA	Upregulated in tumor tissue
miR-584	UUAUGGUUUGCCUGGGACUGAG	Upregulated in tumor tissue
miR-1	UGGAAUGUAAAGAAGUAUGUAU	Downregulated in tumor tissue
miR-143	UGAGAUGAAGCACUGUAGCUC	Downregulated in tumor tissue
miR-145	GUCCAGUUUUCCCAGGAAUCCCU	Downregulated in tumor tissue
miR-192	CUGACCUAUGAAUUGACAGCC	Downregulated in tumor tissue
miR-194	UGUAACAGCAACUCCAUGUGGA	Downregulated in tumor tissue
miR-195	UAGCAGCACAGAAAUAUUGGC	Downregulated in tumor tissue
miR-215	AUGACCUAUGAAUUGACAGAC	Downregulated in tumor tissue
miR-26a	UUCAAGUAAUCCAGGAUAGGCU	Downregulated in tumor tissue
miR-26b	UUCAAGUAAUUCAGGAUAGGU	Downregulated in tumor tissue
miR-29c	UAGCACCAUUUGAAAUCGGUUA	Downregulated in tumor tissue
miR-30b	UGUAAACAUCCUACACUCAGCU	Downregulated in tumor tissue
miR-30c	UGUAAACAUCCUACACUCUCAGC	Downregulated in tumor tissue
miR-30e	UGUAAACAUCCUUGACUGGAAG	Downregulated in tumor tissue
miR-375	UUUGUUCGUUCGGCUCGCGUGA	Downregulated in tumor tissue
miR-378	ACUGGACUUGGAGUCAGAAGG	Downregulated in tumor tissue
